# From molecular crosstalk to precision therapy: targeting ferroptosis and cuproptosis in oral squamous cell carcinoma

**DOI:** 10.3389/fonc.2026.1808762

**Published:** 2026-04-30

**Authors:** Xikun Ma, Yiqi Chen, Huaqing Mai, Pengyu Lai, Adili Alimujiang, Mingxing Lu

**Affiliations:** 1 School of Stomatology, Jinan University, Guangzhou, China; 2 State Key Laboratory of Oral & Maxillofacial Reconstruction and Regeneration, Key Laboratory of Oral Biomedicine Ministry of Education, Hubei Key Laboratory of Stomatology, School & Hospital of Stomatology, Wuhan University, Wuhan, China

**Keywords:** cuproptosis, ferroptosis, nano-drug delivery systems, oral squamous cell carcinoma, programmed cell death

## Abstract

Oral squamous cell carcinoma (OSCC) is one of the most common malignant tumors in the head and neck region, where conventional therapies have limited efficacy and patients have poor prognosis. As newly identified metal ion-dependent programmed cell death modalities, ferroptosis and cuproptosis play critical roles in tumor metabolism, immune microenvironment remodeling, and therapeutic resistance, representing emerging research foci in OSCC. This review examines the core molecular mechanisms of ferroptosis and cuproptosis and delineates their respective roles in OSCC initiation, progression, immune evasion, and therapeutic resistance. Furthermore, we explore the crosstalk between these two cell death modalities across oxidative stress, metabolic, and signaling networks. Synthesizing these findings, we outline emerging combination strategies that concurrently target ferroptosis and cuproptosis, and discuss current challenges and future directions for translating these concepts into precision therapies for OSCC.

## 1 Introduction

Oral squamous cell carcinoma (OSCC) ranks among the most prevalent malignant tumors in the head and neck region, characterized by high invasiveness and poor prognosis. In 2022, 389,485 new OSCC cases and 188,230 related deaths were reported worldwide, yielding a case-fatality rate of 48%, and its incidence continues to rise annually (1). Conventional treatments for OSCC primarily consist of surgical resection, radiotherapy, and platinum-based chemotherapy. Despite their widespread use, these regimens are frequently limited by significant treatment-related toxicity, high rates of local recurrence and distant metastasis, frequent development of chemoresistance, and unavoidable damage to adjacent normal tissues. These drawbacks highlight the critical need for novel, more effective, and tumor-specific therapeutic approaches that can improve outcomes while minimizing adverse effects. In recent years, ferroptosis and cuproptosis, as novel forms of programmed cell death, have garnered increasing attention in tumor research. Ferroptosis is characterized by iron-dependent lipid peroxidation, while cuproptosis induces proteotoxic stress through the binding of copper ions to mitochondrial proteins. This review systematically delineates the molecular mechanisms, crosstalk, and therapeutic potential of ferroptosis and cuproptosis in OSCC, providing a theoretical framework for novel combination strategies.

## 2 Research progress on ferroptosis in OSCC

### 2.1 Mechanisms of ferroptosis

**Figure 1:**
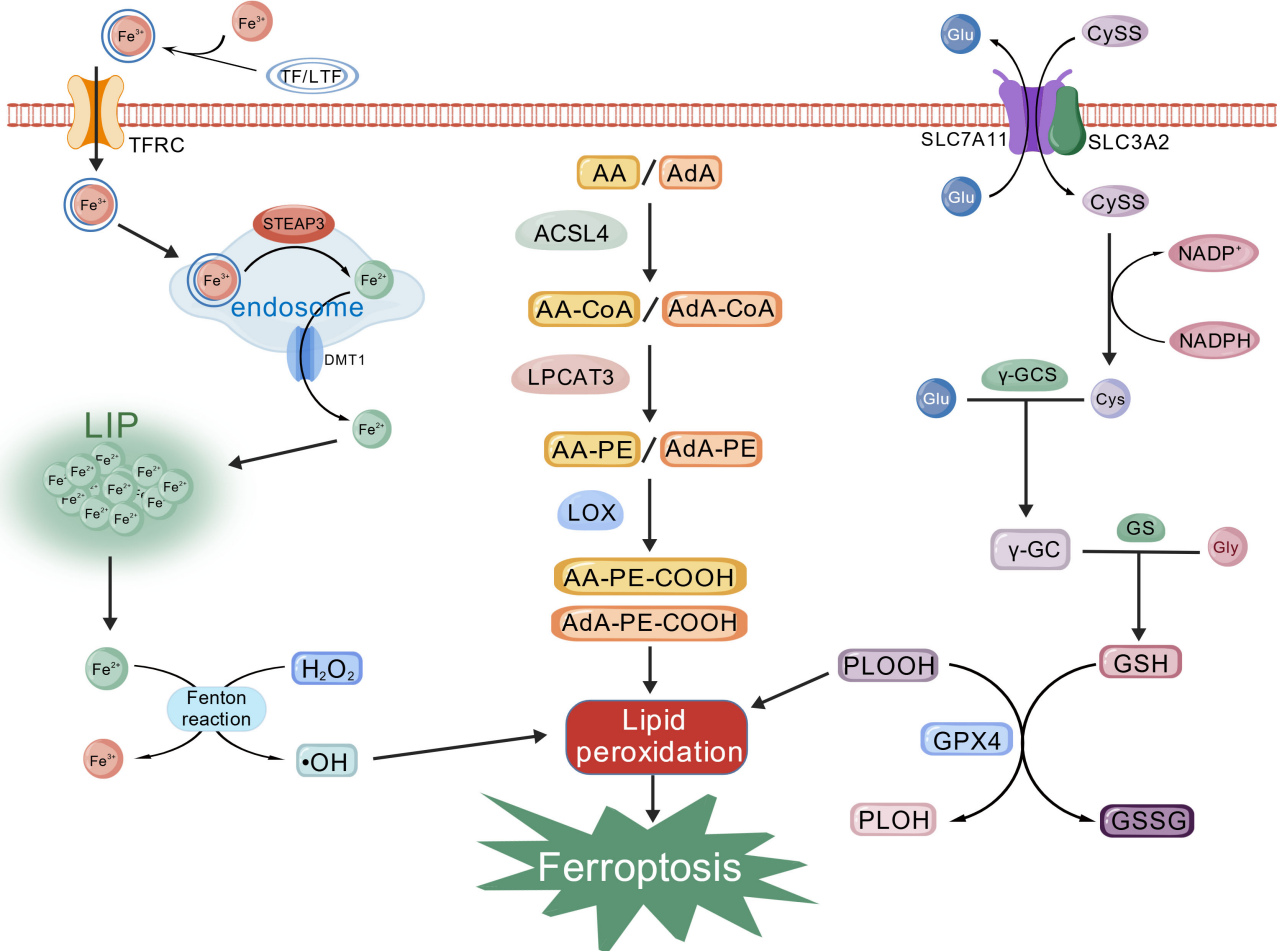
The core mechanisms diagram of ferroptosis. Created with BioGDP.com (74).

Ferroptosis is a regulated form of cell death driven by the iron-dependent accumulation of lethal lipid peroxides. Morphologically it is distinct from apoptosis, necrosis and pyroptosis: mitochondria shrink, their membranes become denser and cristae disappear, whereas nuclear architecture remains intact (2). As shown in **Figure 1**, the core mechanism of ferroptosis comprises three inter-locking events: perturbation of iron metabolism, build-up of lipid peroxides, and collapse of the glutathione–glutathione peroxidase 4 (GSH–GPX4) antioxidant axis.

Execution of ferroptosis requires an expanded labile iron pool (LIP). Extracellular Fe^3+^ bound to transferrin (TF) or lactoferrin (LTF) is imported through transferrin receptor (TFRC). After endocytosis, Fe^3+^ is reduced to Fe^2+^ by Six-Transmembrane Epithelial Antigen of the Prostate 3 (STEAP3) in endosome and subsequently released into the cytoplasmic LIP via divalent metal transporter-1 (DMT1) (3). When iron ion concentration in LIP exceeds physiological thresholds, Fe^2+^ fuels the Fenton reaction to convert H_2_O_2_ into highly toxic hydroxyl radicals (•OH), igniting membrane lipid peroxidation that culminates in cell death (4). Additionally, ferritinophagy mediated by nuclear receptor co-activator 4 (NCOA4) degrades ferritin and liberates redox-active iron, amplifying the lethal signal of ferroptosis (5).

Peroxidation of polyunsaturated fatty acid phospholipids (PUFA-PLs) is the biochemical hallmark of ferroptosis. This process is executed through a dedicated enzymatic cascade. Specifically, free polyunsaturated fatty acids (PUFAs) such as arachidonic acid (AA) and adrenic acid (AdA) are first activated by acyl-CoA synthetase long-chain family member 4 (ACSL4) to form their acyl-CoA esters (AA-CoA and AdA-CoA). These activated intermediates are then incorporated into membrane phosphatidylethanolamines (PEs) by lysophosphatidylcholine acyltransferase 3 (LPCAT3), generating the highly oxidizable species AA-PE and AdA-PE (6). Lipoxygenases (LOXs) subsequently oxidize these PE substrates into phosphatidylethanolamine hydroperoxides (PE-OOHs), which constitute the lethal lipid signals that directly execute ferroptosis (7). Therefore, ACSL4 and LPCAT3 are established positive regulators of ferroptosis, and their cellular expression levels are strong determinants of ferroptosis sensitivity.

The intracellular GSH-GPX4 axis constitutes the core antioxidant defense mechanism against ferroptosis. GPX4 catalyzes the reduction of phospholipid hydroperoxides (PLOOHs) using GSH as a cofactor, thereby generating oxidized glutathione (GSSG) and non-toxic phospholipid alcohols (PLOHs). This reaction terminates the lipid peroxidation chain reaction, thereby preventing membrane damage (8). GSH biosynthesis is sustained by system Xc^−^, a cystine/glutamate antiporter composed of solute carrier family 7 member 11 (SLC7A11) and solute carrier family 3 member 2 (SLC3A2). System Xc^−^ imports extracellular cystine (CySS) in exchange for glutamate (Glu). Intracellular CySS is then reduced to cysteine (Cys) by NADPH. Subsequently, Cys and Glu are conjugated by γ-glutamylcysteine synthetase (γ-GCS) to form γ-glutamylcysteine (γ-GC), which is finally combined with glycine by glutathione synthetase (GS) to produce GSH (9). Downregulation of SLC7A11 or depletion of GSH leads to GPX4 inactivation, resulting in the lethal accumulation of lipid peroxides and triggering ferroptosis (9). Furthermore, ferroptosis suppressor protein 1 (FSP1) has been identified as a glutathione-independent inhibitor of ferroptosis. It operates via the FSP1-CoQ10-NAD(P)H pathway, which cooperates with the GPX4/GSH system to suppress lipid peroxidation and ferroptosis, representing an independent parallel antioxidant system (10).

Accumulating evidence has uncovered two additional parallel pathways that act as critical regulators of ferroptosis. The GCH1/BH4/DHFR axis operates independently of GPX4: guanosine triphosphate cyclohydrolase 1 (GCH1) catalyzes the rate-limiting step in the *de novo* synthesis of tetrahydrobiopterin (BH4) from GTP, producing BH4 as a potent radical-trapping antioxidant (RTA) that directly neutralizes lipid peroxyl radicals (LOO•) and prevents membrane lipid peroxidation (11). Unlike GPX4, which requires GSH as a cofactor, BH4 functions as a membrane-soluble antioxidant that can partition into lipid bilayers to intercept peroxidation chain reactions, while dihydrofolate reductase (DHFR) subsequently regenerates BH4 from its oxidized form (BH2), maintaining this protective shield through continuous recycling (12). The DHODH-CoQ pathway represents another GPX4-independent mechanism: embedded in the mitochondrial inner membrane, dihydroorotate dehydrogenase (DHODH) oxidizes dihydroorotate to orotate while reducing ubiquinone (CoQ) toubiquinol (CoQH_2_) —a lipophilic radical-trapping antioxidant that detoxifies lipid peroxides where GPX4 access is limited. This creates a dual surveillance system: GPX4 protects cytosolic and plasma membrane compartments, while DHODH-CoQH_2_ specifically safeguards mitochondrial membrane integrity (13). However, Subsequent study has challenged this concept by demonstrating that the ferroptosis-sensitizing activity of DHODH inhibitors arises predominantly from off-target inhibition of FSP1, rather than specific blockade of DHODH itself (14). Therefore, the therapeutic potential of targeting DHODH requires cautious interpretation. These findings highlight the remarkable redundancy and spatial complexity of ferroptosis regulation, and underscore the necessity of rigorously distinguishing on-target from off-target effects when exploring novel ferroptosis-inducing strategies for cancer therapy.

Beyond the core mechanisms described above, mounting evidence has revealed that multiple signaling pathways are involved in modulating ferroptosis. The Nrf2/HO-1 pathway suppresses ferroptosis by upregulating antioxidant enzymes (15). Conversely, p53 promotes ferroptosis by repressing the expression of SLC7A11 (16). Additionally, the PI3K/AKT/mTOR metabolic signaling pathway has been shown to indirectly sensitize cells to ferroptosis (17). These regulatory mechanisms provide novel insights for developing targeted therapeutic strategies against ferroptosis.

### 2.2 Ferroptosis in OSCC: tumor biology, immune modulation, and therapy resistance

Research on ferroptosis in OSCC began only recently, yet it has rapidly become a focal point at the intersection of tumor metabolism and cell-death biology. Accumulating *in-vitro* and *in-vivo* data show that ferroptosis not only participates in OSCC initiation and progression but is also closely associated with immune escape, chemoresistance, and poor prognosis.

Ferroptosis plays a fundamental role in OSCC biology. On one hand, OSCC tissues universally display a ferroptosis-resistant phenotype typified by up-regulated GPX4 and high SLC7A11 expression (18). On the other hand, pharmacological induction of ferroptosis markedly suppresses the proliferation, migration, and invasion of OSCC cells. The two major classes of ferroptosis inducers—system Xc^−^ inhibitors and GPX4 inhibitors—have both been validated in OSCC models. In a study by Zhou et al. (19), the system Xc^−^ inhibitor sulfasalazine (SSZ) was applied to human tongue squamous cell carcinoma CAL27 cells and their cisplatin-resistant counterparts (CAR). SSZ was found to suppress the transcription factor AEBP1, thereby down-regulating its direct targets GPX4 and SLC7A11. This led to GSH depletion, a burst of lipid peroxides, and accumulation of labile iron, collectively triggering ferroptosis. All these effects were reversed by the iron chelator deferoxamine (DFO), confirming their iron dependency. Liu et al. (20) extended these findings to radioresistant OSCC models. They demonstrated that the GPX4 inhibitor RAS-selective lethal 3 (RSL3) downregulated GPX4 expression, elevated lipid peroxidation and free iron levels, restored the cytotoxicity of X-ray radiation, and delayed tumor growth. The ferroptosis inhibitor ferrostatin-1 (Fer-1) abolished this radiosensitizing effect, indicating that GPX4 inhibition holds promise as a ferroptosis-based strategy for radiosensitization in OSCC.

Beyond directly killing tumor cells, ferroptosis also remodels the tumor immune microenvironment. Lipid peroxides released early during ferroptosis act as damage-associated molecular patterns (DAMPs), promoting dendritic cell (DC) maturation and antigen cross-presentation, which in turn primes CD8^+^ T cells and enhances anti-tumor immunity (21). Genetic or pharmacologic GPX4 deletion cripples the antioxidant shield of cancer cells, amplifies this ferroptosis-driven immune loop, and sensitizes tumors to immune-checkpoint blockade (ICB). Multiple pre-clinical studies have shown that GPX4 inhibitors or GPX4 knockout synergize with anti-PD-1/PD-L1 antibodies in restoring T-cell activity (22), offering a rational combination strategy for immune-evasive tumors like OSCC. Further supporting this paradigm, Xie et al. (23) identified cadherin-4 (CDH4) as a key immune-escape molecule that is overexpressed in OSCC. Their study demonstrated that CDH4 knockdown inhibited GPX4 activity, induced ferroptosis, and enhanced the infiltration of CD8^+^ T cells and DCs, thereby positioning CDH4 as a promising target for combination therapies leveraging ferroptosis and immunotherapy.

Ferroptosis resistance is a key mechanism underlying chemotherapy resistance in OSCC. Cisplatin-resistant OSCC cells suppress ferroptosis through upregulation of the SLC7A11-GSH-GPX4 axis, while reactivating ferroptosis can restore cisplatin sensitivity and reverse the resistant phenotype (24). Wang L et al. (25) demonstrated that melatonin plus the system Xc^−^ inhibitor erastin promotes ferroptosis via a ROS-dependent mechanism, enhances cisplatin cytotoxicity in OSCC cells, and exhibits no overt toxicity *in vivo*, providing a clinically translatable approach to overcome cisplatin resistance. Wang S et al. (26) uncovered an additional regulatory layer: circular RNA plasmacytoma variant translocation 1 (circPVT1) sponges miR-143-3p to relieve repression of SLC7A11, thereby blocking ferroptosis and accelerating tumor growth. Silencing or knockdown of circPVT1 reinstated ferroptosis and suppressed OSCC progression, highlighting circRNAs as novel modulators of ferroptosis circuitry.

### 2.3 Targeting ferroptosis to treat OSCC

Over the past few years, pharmacological induction of ferroptosis has emerged as a promising anti-cancer paradigm and is now being aggressively translated to OSCC. Current research efforts are primarily concentrated on three strategic fronts: natural products, nano-drug delivery systems (NDDS), and combination chemo-/radiotherapy regimens, all aimed at potentiating ferroptosis induction and overcoming tumor resistance.

Owing to their multi-target activity, low toxicity, and oral availability, natural products have moved to the forefront of ferroptosis research in OSCC. **Table 1** summarizes several popular natural products and their mechanisms. For instance, Wang et al. (27) reported that piperlongumine (PL) silences GPX4, SLC7A11, and ferritin heavy chain 1 (FTH1) while up-regulating the iron importer DMT1. This leads to cytosolic Fe^2+^ overload and the collapse of GSH-dependent antioxidant defenses. Co-administration of PL with the glutaminase inhibitor CB-839 synergistically amplifies ferroptosis and halts OSCC proliferation. Mao et al. (28) showed that resveratrol triggers ferroptosis through the p53/SLC7A11 axis: nuclear translocation of p53 represses SLC7A11 transcription, which cripples CySS uptake and GSH synthesis, ultimately provoking Fe^2+^ accumulation and lipid peroxidation. Zhu et al. (29) demonstrated that quercetin down-regulates SLC7A11 by inhibiting the mTOR/S6K/P70 pathway, thereby lowering GSH levels and boosting lipid peroxides to execute ferroptosis in OSCC cells. Furthermore, curcumin (30), celastrol (31), and dihydroartemisinin (32) have exhibited ferroptosis-inducing capacity in other tumor models and await validation in OSCC (**Table 1**). Collectively, these studies provide a versatile natural-product toolbox that can be rationally combined for precision ferroptosis therapy.

**Table 1:** Natural products that induce ferroptosis.

Natural products	Tumor models (cell lines)	Mechanisms of action	Potential for OSCC therapy
Piperlongumine (27)	OSCC (HSC-3, H400)	Inhibits the expression of SLC7A11, GPX4 and FTH1; increases DMT1, ROS, MDA and Fe^2+^ levels; decreases GSH levels, thereby inducing ferroptosis	Piperlongumine induces ferroptosis in OSCC cells, and this effect is further enhanced when combined with the glutaminase-1 inhibitor CB-839
Resveratrol (28)	OSCC (CAL-27, SCC-9)	Promotes p53 nuclear translocation; inhibits the transcription and expression of SLC7A11; decreases GPX4 and GSH levels; increases Fe^2+^, ROS and LDH levels, thus inducing ferroptosis	Resveratrol can accelerate ferroptosis in OSCC cells by regulating p53/SLC7A11
Quercetin (29)	OSCC (CAL-27, SCC-9)	Inhibits the mTOR/S6K1-P70 pathway; downregulates the expression of GPX4 and SLC7A11; increases ROS and Fe^2+^ levels, leading to mitochondrial dysfunction and ferroptosis	Quercetin induces ferroptosis in OSCC cells by targeting the mTOR/S6K P70 cascade, and this effect is potentiated by mTOR inhibitors.
Curcumin (30)	Colorectal Cancer (SW620, LoVo)	Inhibits the SLC7A11/GSH/GPX4 axis; upregulates p53; downregulates the expression of SLC7A11 and GPX4, inducing ferroptosis	Curcumin induces ferroptosis via the p53–SLC7A11/GPX4 axis, a mechanism readily applicable to OSCC and offering a low-toxic, natural therapeutic strategy
Celastrol (31)	Hepatocellular Carcinoma (Huh-7)	Binds and degrades RRM2, inhibits mTOR phosphorylation, up-regulates TFR1, down-regulates xCT/GPX4, increases ROS/MDA, decreases GSH-Px, induces ferroptosis	Celastrol activates ferroptosis by targeting the RRM2–mTOR axis; this mechanism is equally critical in OSCC and provides a novel strategy for Celastrol-based OSCC therapy.
Dihydroartemisinin (32)	Cervical cancer (HeLa, SiHa)	Inhibits GPX4, triggers NCOA4-mediated ferritinophagy, elevates LIP/Fe^2+^ and lipid ROS; synergizes with Doxorubicin to enhance ferroptosis	Dihydroartemisinin induces ferritinophagy-dependent ferroptosis through NCOA4-driven iron release and GPX4 depletion, a mechanism applicable to OSCC that supports Dihydroartemisinin-based therapeutic strategies.

MDA, Malondialdehyde; LDH, Lactate Dehydrogenase; GSH-Px, Glutathione Peroxidase.

The rapid development of NDDS has increasingly highlighted their potential to enhance ferroptosis induction for the treatment of OSCC. Nanotechnology offers precise spatiotemporal control over ferroptosis inducers, simultaneously improving tumor targeting and minimizing systemic toxicity. Zhao et al. (33) engineered an acid-responsive iron-based nanocomposite by entrapping ultra-small Prussian blue nanoparticles (USPBNPs) within mesoporous calcium silicate nanosphere (MCSN). In the acidic tumor microenvironment of OSCC, this system selectively releases Fe^2+^, which fuels Fenton reactions, evokes robust lipid peroxidation, and significantly suppresses tumor cell proliferation without notable toxicity. Li et al. (34) constructed a photothermal-responsive multifunctional MnO_2_ nanozyme by co-encapsulating the ferroptosis inducer RSL3 inside hollow mesoporous MnO_2_ (HM-MnO_2_) nanoparticles. The nanosystem is guided to OSCC lesions via surface modification with iron-doped polydopamine (Fe-PDA) and the tumor-targeting peptide cRGD. Upon near-infrared laser irradiation, it exhibits multi-enzymatic activity that generates abundant ROS and exhausts GSH. This process triggers ferroptosis, synergizes with photothermal therapy (PTT) by downregulating key ferroptosis suppressors GPX4 and FSP1, induces mitochondrial damage, and ultimately achieves complete tumor ablation. Zhu et al. (35) co-loaded the photosensitizer Ce6 and the ferroptosis inducer erastin into nanoparticles approximately 150 nm in size, constructing a Ce6-erastin nanomedicine system. This formulation accumulates in CAL-27 cells, where it inhibits the expression of SLC7A11 and GPX4 to activate ferroptosis. Concurrently, it supplies oxygen via Fenton reactions, alleviating tumor hypoxia and thereby significantly enhancing the efficacy of Ce6-mediated photodynamic therapy (PDT), resulting in synergistic ferroptosis-PDT. These studies provide novel insights into utilizing nanodelivery systems for multimodal synergistic induction of ferroptosis. In the future, by integrating intelligent responsiveness, immune modulation, and personalized design, ferroptosis nanotherapies can be advanced toward precise diagnosis, treatment, and clinical translation in OSCC.

Combining ferroptosis inducers with conventional chemotherapy or radiotherapy represents a promising strategy to achieve synergistic effects and improve therapeutic outcomes in OSCC. Cisplatin, when combined with Erastin, exhibits enhanced cytotoxicity. Erastin irreversibly inhibits system Xc^−^, leading to sustained GSH depletion and amplification of cisplatin-induced oxidative damage, which ultimately triggers ferroptosis and overcomes cisplatin resistance (36). This synergistic effect has been systematically validated in head and neck squamous cell carcinoma models (37). Radiotherapy (RT) itself can induce ferroptosis by elevating ROS, upregulating ACSL4, and suppressing the SLC7A11/GPX4 axis. The combination of RT with ferroptosis inducers such as RSL3 or Erastin further intensifies lipid peroxidation and increases radiosensitivity, a synergy that has been confirmed in various cancer models (38); further mechanistic and translational studies in OSCC-specific models are warranted. Future research should focus on exploring the potential of integrating ferroptosis inducers with conventional chemo-radiotherapy in OSCC, optimizing combination regimens to improve patient survival. It is crucial to note that ferroptosis inducers carry potential toxicity to normal tissues (39): while inducing ferroptosis in tumor cells exerts anti-cancer effects, non-selective activation inevitably damages normal cells and contributes to the pathogenesis of diverse diseases, including cardiovascular disorders (atherosclerosis, heart failure, myocardial infarction, ischemia-reperfusion injury), neurodegenerative diseases (Alzheimer’s disease, Parkinson’s disease, Huntington’s disease, and Amyotrophic Lateral Sclerosis), stroke, and hematological malignancies. These detrimental effects arise from the non-discriminatory nature of iron-dependent lipid peroxidation and ROS accumulation in healthy tissues. Therefore, careful attention must be paid to dosing, safety, and the development of targeted delivery strategies in clinical applications.

To date, no completed or published clinical trials of ferroptosis-targeted therapy have been reported in OSCC. All relevant evidence remains at the preclinical stage, including studies based on *in vitro* cell lines, patient-derived organoids, and mouse xenograft models. Nevertheless, several investigations have provided rigorous preclinical validation for ferroptosis-targeted strategies in OSCC. A preclinical study by Zhou et al. (40) demonstrated that combining SSZ with anti-IL-1β monoclonal antibody reversed immune suppression, enhanced CD8^+^T-cell infiltration, and amplified ferroptosis-mediated antitumor effects *in vitro* and in a chemically induced OSCC rat model. This combination represents a clinically translatable approach that enhances ferroptosis while remodeling the tumor immune microenvironment. In addition, fat mass and obesity-associated protein (FTO, an m^6^A demethylase) has been identified as a positive regulator of ferroptosis in OSCC. Wang et al. (41) reported that FTO downregulates the anti-ferroptotic proteins ACSL3 and GPX4 by reducing mRNA stability through m^6^A modification. Elevated FTO expression sensitizes OSCC cells to ferroptosis both *in vitro* and *in vivo*. Targeting FTO-mediated epigenetic regulation may therefore represent a novel strategy to enhance ferroptosis-based therapy, particularly in OSCC with low ferroptosis sensitivity.

While significant advances have been made in ferroptosis research in OSCC, another novel metal ion-dependent form of cell death—cuproptosis—has increasingly demonstrated its critical role in OSCC in recent years. Distinct from ferroptosis, cuproptosis is driven by copper ion-induced proteotoxic stress within the mitochondrial respiratory chain. The following section will systematically outline the unique mechanisms of cuproptosis and its current research landscape in OSCC.

## 3 Research progress on cuproptosis in OSCC

### 3.1 Mechanisms of cuproptosis

**Figure 2:**
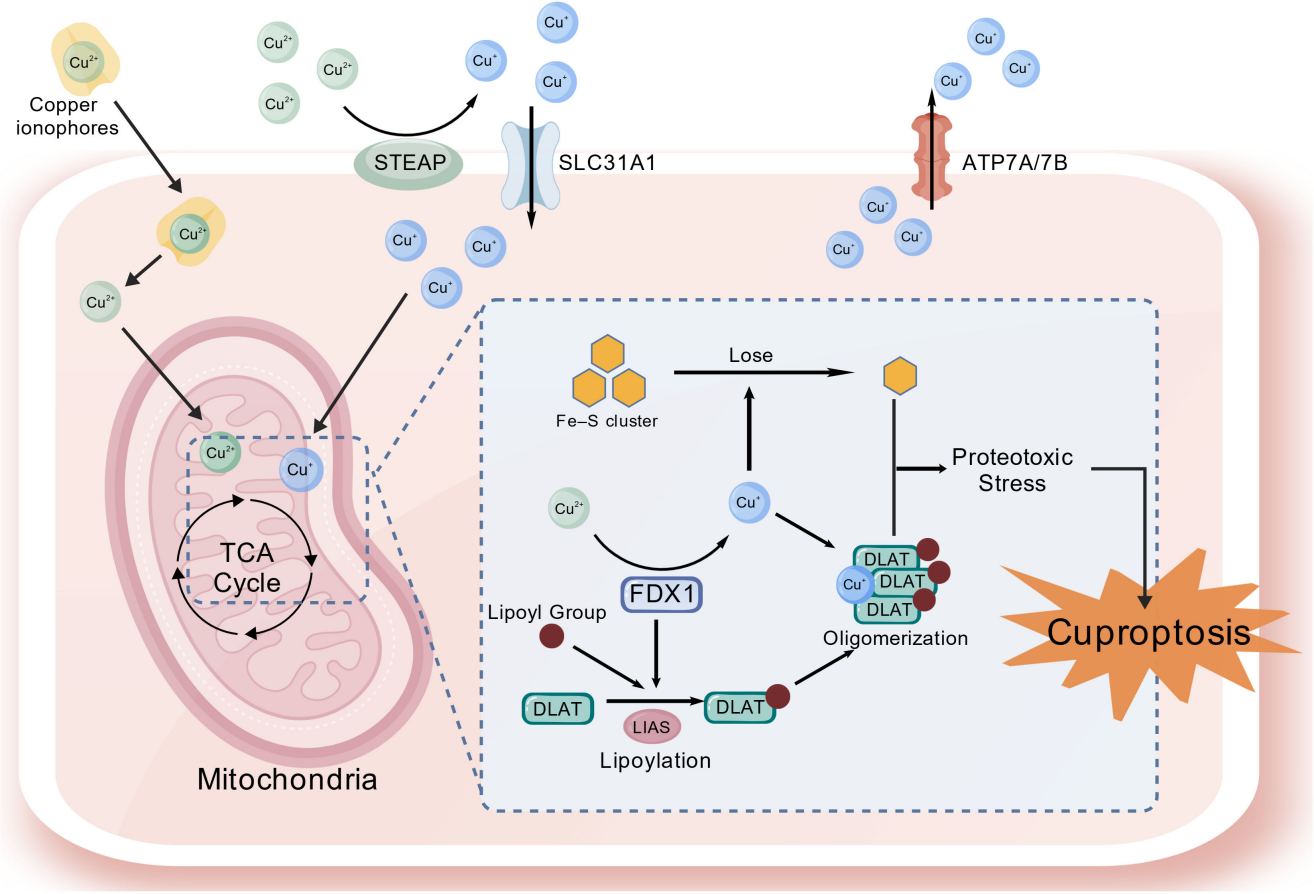
The core mechanisms diagram of cuproptosis. Created with BioGDP.com (74).

Cuproptosis is a newly recognized form of regulated cell death triggered by excessive intracellular copper. Its execution is tightly linked to mitochondrial metabolism. Tsvetkov et al. (42) first coined the term and demonstrated that when copper accumulates inside mitochondria it directly binds lipoated components of the tricarboxylic acid (TCA) cycle, forcing their aberrant oligomerization and the loss of iron–sulfur (Fe–S) cluster proteins. The resulting proteotoxic stress and mitochondrial failure culminate in cell death. The core circuitry is illustrated in **Figure 2**.

Copper overload is the initiating event. Under physiological conditions, extracellular Cu^2+^ is reduced to Cu^+^ by reductases such as STEAP, imported by copper transporter 1 (CTR1, also termed SLC31A1), and then escorted to specific sub-cellular destinations by dedicated chaperones. When cytosolic copper rises, the Cu-ATPases ATP7A and ATP7B translocate from the trans-Golgi network (TGN) to the plasma membrane and pump the surplus copper out of the cell. During copper scarcity, CTR1 is up-regulated and the ATPases return to the TGN, closing a negative-feedback loop that maintains copper homeostasis (43). CTR1 over-expression or functional impairment of ATP7A/7B breaks this loop, allowing copper to accumulate and penetrate mitochondria, thereby priming cuproptosis.

Copper ionophores such as elesclomol accelerate cuproptosis by transporting extracellular Cu^2+^ into the cytoplasm. Once inside the mitochondrial matrix, Cu^2+^ is reduced to the more reactive Cu^+^ by ferredoxin-1 (FDX1) (44). FDX1 also acts as a key reductase, providing electrons to lipoic acid synthase (LIAS) for the synthesis of lipoic acid, which is essential for the lipoylation of dihydrolipoamide S-acetyltransferase (DLAT) and other TCA cycle enzymes (45). Thus, FDX1 occupies a central role in cuproptosis by enabling both copper reduction and protein lipoylation. Excess Cu^+^ binds to the lipoyl-lysine residues of DLAT, promoting its oligomerization and functional disruption, which impairs the pyruvate dehydrogenase complex and compromises TCA cycle activity, leading to proteotoxic stress and mitochondrial dysfunction (46). Furthermore, copper overload indirectly disrupts the biogenesis of mitochondrial Fe–S cluster proteins, which are essential for electron transport chain integrity and metabolic function, thereby exacerbating mitochondrial failure and oxidative stress (47).

Cuproptosis is further modulated by GSH and p53. By acting as a copper chelator, GSH binds mitochondrial Cu^+^ into an inert complex, thereby blocking its interaction with lipoylated proteins (48). Wild-type p53 may enhance susceptibility to cuproptosis by trans-activating genes involved in Fe–S cluster biogenesis (e.g., FDXR) and by rewiring cellular metabolism toward oxidative phosphorylation, thereby increasing reliance on mitochondrial respiration (49). Thus, strategies that either deplete GSH or activate wild-type p53 could sensitize cancer cells to cuproptosis, particularly in combination with copper ionophores.

### 3.2 Cuproptosis in OSCC: from tumor suppression to immune landscape shaping

Although research on cuproptosis in OSCC is still nascent, accumulating evidence underscores its pivotal role in regulating core malignant behaviors of OSCC cells, such as proliferation, apoptosis, migration, and invasion. Yu et al. (50) demonstrated that the circadian clock protein PER2 binds to the C-terminal domain of heat-shock protein 70 (HSP70), thereby displacing AKT from the HSP70–AKT complex and facilitating AKT ubiquitination and degradation. The resulting AKT degradation relieves its suppression on cuproptosis-related gene expression, leading to upregulation of DLAT, PDHB, and SLC31A1, which are essential for cuproptosis execution. This cascade promotes mitochondrial copper accumulation, DLAT oligomerization, and ultimately cuproptosis, significantly suppressing OSCC cell proliferation and tumor growth. Furthermore, activating transcription factor 3 (ATF3) transcriptionally up-regulates PER2, amplifying this lethal cascade. Notably, combining an ATF3 inducer with the copper ionophore elesclomol showed potent synergistic antitumor activity *in vivo*, identifying the “ATF3–PER2–AKT–cuproptosis” axis as a promising therapeutic target for OSCC. Separately, Shen et al. (51) investigated lipoyltransferase 1 (LIPT1), a key gene involved in cuproptosis. They found that LIPT1 expression was significantly lower in OSCC tissues than in adjacent normal mucosa. Notably, patients with lower LIPT1 expression exhibited better overall survival, suggesting that reduced LIPT1-mediated mitochondrial lipoylation may enhance susceptibility to copper-induced stress and suppress tumor progression.

Beyond its direct tumoricidal role, cuproptosis actively sculpts the tumor immune microenvironment in OSCC. By integrating bulk and single-cell RNA-sequencing data, Yuan et al. (52) stratified OSCC into two distinct immune phenotypes: cuproptosis-C1 and cuproptosis-C2. Cuproptosis-C2 tumors were enriched for immune-effector cells, including CD8^+^ T cells, natural killer (NK) cells, γδ T cells, and plasmacytoid dendritic cells (pDCs), consistent with a cytotoxic immune profile. In contrast, cuproptosis-C1 tumors were characterized by an abundance of T helper cells, central memory T (Tcm) cells, and Th2 cells, alongside elevated expression of immune-checkpoint molecules—a profile that may be associated with immune exhaustion and potentially greater responsiveness to immune-checkpoint blockade (ICB), though this remains to be functionally validated. This work underscores cuproptosis gene signatures as key determinants of the immune landscape and therapeutic vulnerability in OSCC. In another study, Kong et al. (53) developed a prognostic risk model based on four cuproptosis-related long non-coding RNAs (CRLs). Patients in the high-risk group exhibited down-regulated immune-effector pathways, elevated expression of the checkpoint molecules PD-L1 and LAG-3, and a trend toward an immune-evasive phenotype, suggesting potential resistance to immunotherapy. Consequently, CRL signatures not only predict patient outcome but may also inform the selection of combinatorial immunotherapy regimens. Expression patterns of cuproptosis-related genes or lncRNAs mirror the abundance of tumor-infiltrating immune cells, the propensity for immune evasion, and the likely response to immunotherapy, positioning them as promising biomarkers for predicting immunotherapy efficacy and guiding combination strategies in OSCC.

Altered expression of cuproptosis-related genes is closely linked to chemosensitivity in OSCC. An OSCC subgroup termed Epi_2 over-expresses the cuproptosis repressor CDKN2A and displays heightened glycolysis, reinforced glutathione metabolism, and activated hypoxia signaling, which collectively enable the tumor to evade cuproptosis. Clinically, Epi_2 tumors are associated with lymph-node metastasis and refractoriness to both immune-checkpoint blockade and anti-EGFR therapies (54), implying that therapeutic targeting of cuproptosis escape mechanisms could reverse such resistance. Separately, the copper-exporting ATPase ATP7B has been implicated in cisplatin resistance via small extracellular vesicles (sEVs). Cisplatin-resistant cells with high ATP7B expression secrete sEVs enriched in heat-shock protein 90 (HSP90) and epithelial cell adhesion molecule (EpCAM). Upon uptake by drug-sensitive cells, these vesicles up-regulate ATP7B, reduce intracellular copper, and thereby impart a resistant phenotype. Notably, the sEV generation inhibitor GW4869 suppresses ATP7B and the copper chaperone Atox1, restoring cisplatin-induced apoptosis (55). This highlights the ATP7B/sEV axis as a promising additional avenue for overcoming chemotherapy resistance.

Collectively, cuproptosis constitutes a tripartite regulatory circuit—tumor suppression, immune modulation, and drug sensitivity—in OSCC. Target optimization, rational combination regimens, and dynamic biomarkers built around this pathway may break current therapeutic ceilings and deliver precise, durable clinical benefit.

### 3.3 Targeting cuproptosis to treat OSCC

Current therapeutic strategies targeting cuproptosis in OSCC revolve around three main approaches: exploiting copper ionophores, engineering NDDS, and designing combination regimens with conventional therapies. Copper ionophores demonstrate unique advantages in inducing cuproptosis, thereby positioning them as promising novel anticancer agents for the treatment of OSCC. The two most clinically advanced candidates are elesclomol (ES) and disulfiram (DSF). ES conveys copper to the mitochondrial matrix, where the metal ions template the lipoylation-dependent aggregation of TCA-cycle enzymes (e.g., DLAT) and precipitate cuproptosis. Its cytotoxicity is strictly proportional to mitochondrial respiratory activity, making it exquisitely potent against metabolically hyperactive or drug-refractory sub-clones (56). DSF—an old anti-alcoholism drug—has recently been repurposed as a copper ionophore that triggers cuproptosis in a wide spectrum of tumors, with particular efficacy against stem-like or metabolically aggressive populations (57). Although OSCC-focused data are still scarce, the tumor type is characterized by heightened mitochondrial metabolism, elevated oxidative stress and intrinsic cisplatin resistance (58), all of which constitute a biochemical signature predictive of cuproptosis vulnerability. Exploiting the mechanistic paradigms established with ES and DSF in other cancers should therefore provide a rational blueprint for precision therapy in OSCC.

Although systemic induction of cuproptosis shows potential for cancer therapy, off-target toxicity and systemic adverse effects remain unavoidable. Nonspecific disruption of copper homeostasis disrupts normal mitochondrial respiration, redox balance, and metalloenzyme function in healthy tissues, leading to organ damage. Systemically administered cuproptosis inducers lack strict tumor targeting and accumulate in liver, kidney, and nervous tissues, causing toxicity (59). This challenge is exemplified by the early clinical setbacks of ES, which failed to improve overall survival, underscoring the critical need for precise patient stratification based on biomarkers such as mitochondrial metabolic capacity. Similarly, the repurposed drug DSF faces translational hurdles due to its complex pharmacokinetics and the instability of its bioactive copper complex, which complicates reliable tumor targeting. Consequently, the development of NDDS is viewed as an indispensable strategy. Such systems are designed to circumvent these limitations by enabling targeted delivery and controlled release of copper, thereby maximizing intratumoral cuproptosis induction while minimizing systemic toxicity.

NDDS enable targeted delivery and controlled release of copper ions, thereby precisely inducing cuproptosis to enhance therapeutic efficacy while minimizing toxicity. Furthermore, their integration with immunotherapy strategies can establish a multimodal synergistic therapeutic framework. Proof-of-concept studies across different cancer models validate this approach. Li et al. (60) engineered a tumor-microenvironment-responsive copper-based metal-organic framework (Cu-MOF) co-loaded with Cu^2+^ and the fatty-acid-metabolism inhibitor orlistat (ORL). The resulting ORL@Cu-MOF simultaneously triggered cuproptosis and blocked lipid reprogramming, which markedly suppressed primary OSCC growth and cervical lymph-node metastasis while remodeling the immune milieu via up-regulation of PD-1. Its combination with an anti-PD-1 antibody (αPD-1) elicited a potent systemic anti-tumor response, offering a directly testable cuproptosis-immunotherapy platform for OSCC. In a separate study, Guo et al. (61) developed ROS-responsive nanoparticles (NP@ESCu) for the co-delivery of Cu^2+^ and ES. Cuproptosis induction coupled with PD-L1 up-regulation sensitized bladder tumors to αPD-1, validating this combinatorial concept in a solid-tumor setting and warranting its extension to OSCC. Further expanding the toolkit, Wang et al. (62) constructed a near-infrared (NIR)-activatable copper-delivery patch (CuD@PM) integrated with microneedles loaded with tri-acetylated azacitidine (TAc-AzaC). In HNSCC models, on-demand photothermal release of Cu^+^ triggered cuproptosis, expanded CD8^+^ T-cell populations, and suppressed regulatory immune cells, achieving safe and efficient tumor ablation—an approach readily adaptable to OSCC. Moving forward, future efforts should focus on refining stimulus-responsive copper nanocarriers, defining optimal sequencing with immunotherapies, and validating predictive biomarkers to facilitate the seamless integration of cuproptosis-based regimens into existing OSCC treatment algorithms.

Currently, no clinical trials targeting cuproptosis have been reported in OSCC. All relevant progress remains in the preclinical stage, yet several studies have demonstrated the translational potential of cuproptosis-directed interventions. Previous work has demonstrated that the copper ionophore elesclomol effectively induces mitochondrial copper accumulation and triggers cuproptosis in OSCC. When combined with a PER2-activating agent, this strategy further enhances antitumor efficacy through synergistic cuproptosis induction (50). Emerging evidence also indicates that the copper transporter ATP7B promotes cisplatin efflux and the secretion of sEVs in OSCC, thereby reducing the effective intracellular drug concentration, suppressing cuproptosis, and contributing to cisplatin resistance (55). Targeting ATP7B-mediated copper efflux and sEV secretion thus represents a promising approach to restore cuproptosis sensitivity and overcome chemoresistance in refractory OSCC.

As an emerging oncotherapeutic strategy, cuproptosis-directed therapy holds broad promise for OSCC, and future exploration should therefore reach beyond ionophores and nanomedicines to encompass systematic mining of natural-product libraries for plant-derived cuproptosis inducers, structure-based design of next-generation agents that selectively engage FDX1, DLAT or ATP7B, and rational integration of cuproptosis activators with radiation or cisplatin so as to override canonical resistance pathways.

Ferroptosis and cuproptosis are mechanistically distinct: the former relies on Fe^2+^-dependent lipid peroxidation, whereas the latter is driven by Cu^+^-induced mitochondrial protein aggregation. Despite these differences, both are forms of metal ion-dependent regulated cell death and may exhibit close crosstalk within regulatory networks, metabolic pathways, and oxidative stress responses based on emerging evidence. Therapeutically, targeting ferroptosis focuses on damaging cell membrane lipids, while targeting cuproptosis centers on inducing mitochondrial proteotoxicity. Their modes of cell death are complementary rather than redundant, suggesting potential for synergistic induction. The following sections will systematically elaborate on the proposed crosstalk mechanisms between ferroptosis and cuproptosis and explore corresponding synergistic therapeutic strategies, noting that many of these interactions require further experimental validation.

## 4 Crosstalk between ferroptosis and cuproptosis

**Figure 3:**
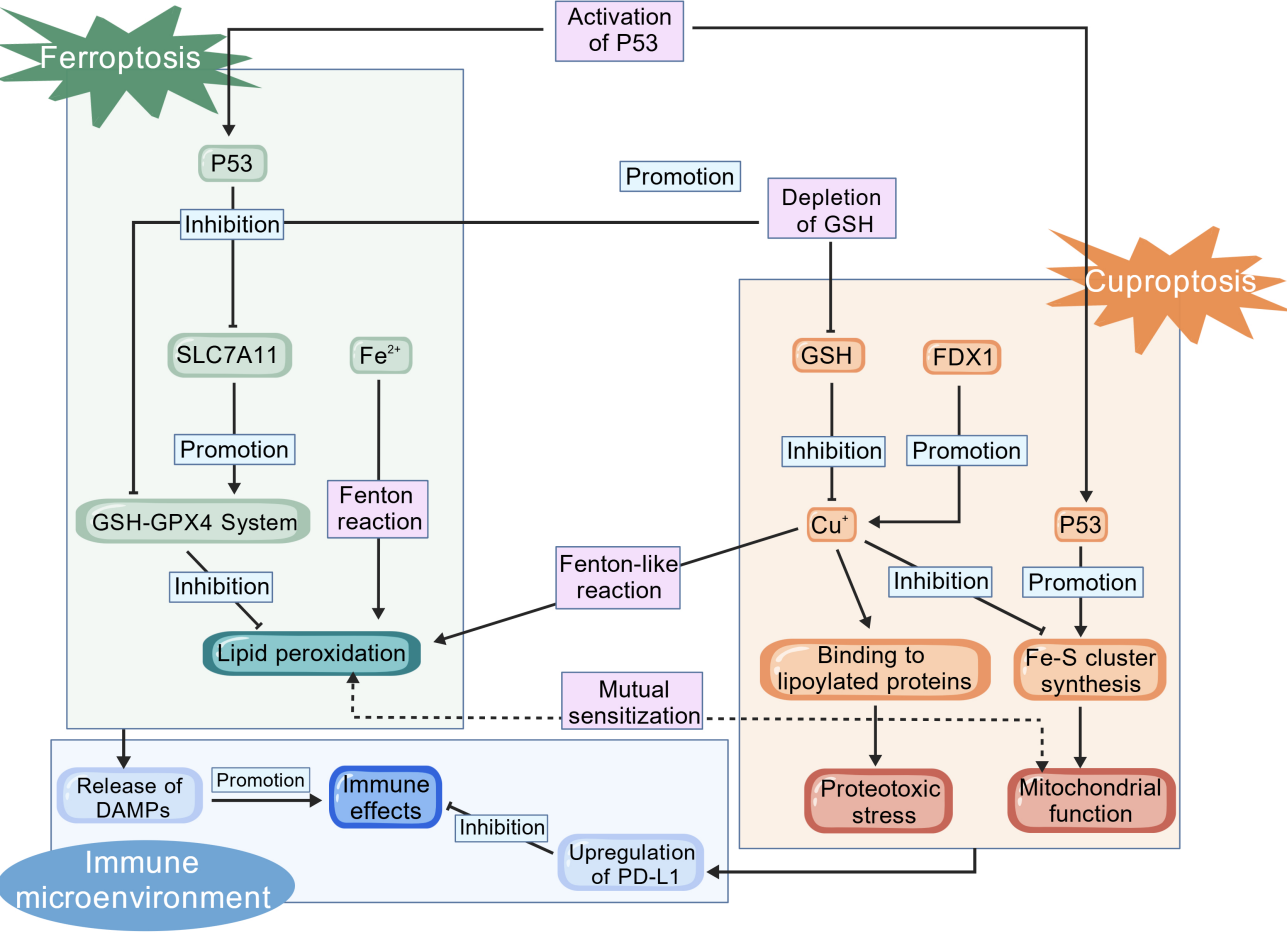
The crosstalk mechanisms between ferroptosis and cuproptosis. Created with BioGDP.com (74).

Accumulating evidence indicates that the pathways of ferroptosis and cuproptosis converge at several key biological nodes, including oxidative stress, GSH metabolism, mitochondrial function, and p53 regulation. Their integrated circuitry is summarized in **Figure 3**.

### 4.1 A shared oxidative-stress axis

Both ferroptosis and cuproptosis hinge on cellular redox homeostasis. During ferroptosis, Fe^2+^ fuels Fenton reaction to generate •OH, which directly drive lipid peroxidation (4). Gao et al. (63) demonstrated that ES transports Cu^2+^ into mitochondria, promotes the degradation of ATP7A, thereby trapping copper. The accumulated copper then catalyzes a Fenton-like reaction to produce •OH. The ensuing burst of lipid peroxides accelerates ferroptosis, revealing that Cu^+^ can indirectly propagate the ROS-lipid peroxidation cascade of ferroptosis.

### 4.2 GSH as a dual-function rheostat

GSH levels critically influence susceptibility to both ferroptosis and cuproptosis. As the cofactor of GPX4 it neutralizes lipid hydroperoxides; its depletion therefore precipitates ferroptosis (9). Consequently, GSH chelates mitochondrial Cu^+^, prevents copper binding to lipoylated proteins and restrains cuproptosis (48). Zhang et al. (64) demonstrated that disulfiram–copper (DSF/Cu) blocks ubiquitin-dependent degradation of the cystine transporter xCT, leading to GSH exhaustion, lipid-peroxide accumulation and compensatory xCT up-regulation. The net result is simultaneous ferroptosis and cuproptosis; additional xCT inhibition abolishes GSH rebound and potentiates DSF/Cu lethality, identifying GSH as a central molecular cross-road.

### 4.3 Mitochondrial metabolic crosstalk

Mitochondria, as a vital source of cellular energy, serve as a common target for both ferroptosis and cuproptosis. During ferroptosis, mitochondria not only act as a site for lipid peroxidation but also function as a central hub amplifying death signals (65).Wang et al. (66) reported that the ferroptosis inducer erastin compromises mitochondrial respiration, which indirectly elevates intramitochondrial copper levels and thereby sensitizes cancer cells to cuproptosis. While in cuproptosis, the disruption of the TCA cycle and electron transport chain by accumulated copper ions generates profound metabolic stress. This stress heightens cellular susceptibility to lipid peroxidation, ultimately promoting the execution of ferroptosis (67).

### 4.4 P53 as a bi-functional gatekeeper

The tumor suppressor p53 is a pivotal modulator of both ferroptosis and cuproptosis. In ferroptosis, p53 transcriptionally represses SLC7A11, thereby curtailing GSH synthesis and sensitizing cells to this form of death (16). In the context of cuproptosis, p53 orchestrates a metabolic shift by dampening glycolysis while enhancing the TCA cycle and oxidative phosphorylation. Concurrently, it up-regulates genes involved in iron-sulfur (Fe-S) cluster assembly and suppresses SLC7A11 to lower GSH levels. These combined actions augment the cytotoxicity caused by copper-bound lipoylated protein aggregation and Fe-S cluster loss (55). Consequently, pharmacologic restoration or functional mimicry of p53 activity represents a promising strategy to synchronously prime OSCC cells for ferroptosis and cuproptosis.

### 4.5 Synergistic versus antagonistic potential

These mechanistic overlaps provide a compelling rationale for a combinatorial therapeutic assault. Strategies such as combining copper ionophores with ferroptosis inducers, or superimposing DSF/Cu regimens on a background of GSH depletion or p53 activation, could overcome resistance to single-death modalities and deliver a potent ferroptosis-cuproptosis “double punch” to OSCC.

However, the two death pathways imprint fundamentally opposite signatures on the tumor immune microenvironment. Ferroptosis promotes immunogenicity by releasing DAMPs and lipid peroxides that mature dendritic cells and expand CD8^+^ T-cell populations, thereby boosting anti-tumor immunity (21). In stark contrast, cuproptosis can foster an immunosuppressive niche by up-regulating PD-L1 and excluding immune infiltrates, thereby driving immune evasion (68). Consequently, future therapeutic protocols designed to sequentially or simultaneously trigger both death routes must critically interrogate whether the inflammation ignited by ferroptosis can effectively neutralize the immunosuppression orchestrated by cuproptosis. Successfully exploiting this antagonism holds the transformative potential to convert an immune-cold tumor into an immune-hot one, ultimately augmenting the efficacy of immune-checkpoint-based immunotherapy in OSCC. Therefore, the design of combination regimens must consider not only metabolic crosstalk but also the opposing immunological effects. Sequential scheduling—for example, priming with ferroptosis inducers to activate antitumor immunity before introducing cuproptosis-targeted agents—may be required to avoid premature immunosuppression and achieve true synergy.

Therefore, the design of combination regimens must be informed not only by the metabolic crosstalk (e.g., via GSH depletion) but also by real-time immune monitoring of the TME. Sophisticated scheduling, rather than simple concurrent administration, may be required to harness the immunogenic potential of ferroptosis while mitigating any immunosuppressive byproducts of cuproptosis.

While the mechanistic overlaps described above provide a compelling rationale for combinatorial therapeutic approaches, several critical questions remain unresolved. A major limitation is that current evidence for direct molecular crosstalk between ferroptosis and cuproptosis derives from studies in different cancer types or cell line models; whether these interactions operate identically in OSCC-specific contexts requires validation. Another critical unresolved issue is that the immunological consequences of simultaneous ferroptosis-cuproptosis induction in OSCC patients remain largely theoretical. The antagonistic effects observed in preclinical models —ferroptosis promoting immunogenicity versus cuproptosis potentially inducing immunosuppression— may vary significantly across individual tumor microenvironments. Furthermore, biomarkers capable of dynamically monitoring the balance between these death modalities in real-time clinical settings are currently unavailable. Addressing these gaps will require integrated multi-omics analyses, patient-derived organoid models incorporating stromal and immune components, and carefully designed clinical trials with built-in mechanistic endpoints.

## 5 Combined ferroptosis-cuproptosis strategy for OSCC

The extensive crosstalk between ferroptosis and cuproptosis across metabolic and signaling networks furnishes a strong mechanistic rationale for their simultaneous induction. Emerging nanotherapeutic platforms, designed to synchronously deliver metal ions and sensitizing agents, can be categorized by their operational logic: synchronized delivery, endogenous/exogenous responsive release, and controlled sustained release. Different nanodrug systems designed for this purpose, along with their mechanisms of action and immunomodulatory effects, are summarized in **Table 2**.

**Table 2:** Nanodrug systems for synergistic induction of ferroptosis and cuproptosis.

Nanodrug system	Core components	Synergistic mechanism	Immune effects
CuP/Er (69)	Cu^2+^+Erastin	Erastin depletes GSH; Cu activates ICD, upregulates PD-L1, induces DLAT aggregation	Synergizes with ICB to enhance antitumor immunity
CACuPDA (70)	Cu^2+^+CA+PDA	Cu depletes GSH and inhibits GPX4; CA amplifies ROS	Increases CTL infiltration, reduces Tregs, synergizes with ICB
PC@B-H (71)	Cu^2+^+PLB+HA	Cu induces DLAT aggregation; PLB inhibits GPX4	Induces ICD, promotes DC maturation and CTL infiltration
MetaCell (72)	Fe-Cu MOF+ thermosensitive liposome+ Neutrophil	Fe/Cu synergistically catalyze ROS; Cu induces DLAT aggregation	Activates immune response, enhances tumor targeting and retention
NCs-AS-ALG Hydrogel (73)	NCs+AS+ALG	Fe/Cu react with AS to generate •C radicals, amplifying oxidative stress	Activates multiple death pathways, enhances immune memory

PDA, polydopamine; HA, hyaluronic acid; CTL, cytotoxic T lymphocyte; Treg, regulatory T cell.

### 5.1 Synchronized delivery strategy

Synchronized delivery of metal ions and sensitizing drugs via NDDS is an effective strategy for inducing both ferroptosis and cuproptosis. Li et al. (69) engineered CuP/Er, a nanoparticle that co-delivers Cu^2+^ and erastin. Erastin inhibits system Xc^−^ to lower GSH, reverses the Warburg effect, and thus primes mitochondria to amplify copper-mediated cuproptosis, culminating in irreversible mitochondrial damage. The combined insult elicits robust immunogenic cell death (ICD), promotes dendritic cell maturation and T cell infiltration, and synergizes with immune-checkpoint blockade (ICB) to suppress tumor growth and metastasis. Translation to OSCC models and further refinement of particle composition and release kinetics are logical next steps.

The strength of synchronized delivery strategy lies in its ability to consolidate multiple synergistic mechanisms into a single formulation, greatly simplifying the complexity of combination therapy while offering excellent targeting capability and immunostimulatory potential; nevertheless, large-scale manufacture, long-term stability and extended biocompatibility of the nanomedicines remain critical bottlenecks for clinical translation, and tighter control of metal-ion toxicity still needs to be refined.

### 5.2 Endogenous or exogenous responses

Leveraging the tumor microenvironment as an endogenous trigger can further amplify both ferroptosis and cuproptosis. Jiang et al. (70) developed GSH-responsive copper-doped polydopamine nanoparticles (CACuPDA). In GSH-rich tumors, Cu^2+^ and cinnamaldehyde (CA) are liberated; Cu^2+^ consumes GSH and down-regulates GPX4 to provoke ferroptosis, whereas Cu^+^-mediated oligomerization of DLAT drives cuproptosis, accompanied by FDX1 down-regulation. The mutually reinforcing oxidative burst breaks immune-suppressive barriers and sensitizes tumors to ICB. Sun et al. (71) extended this concept to a GSH/pH dual-responsive copper complex (PC@B-H) that releases Cu^2+^ and plumbagin (PLB) inside the acidic, GSH-high OSCC niche. Ferroptosis, cuproptosis and necroptosis are simultaneously activated, achieving 92.3% tumor growth inhibition and durable immune memory.

In addition to endogenous biomarker triggers, external physical cues such as near-infrared light, ultrasound, or magnetic hyperthermia can be exploited as responsive switches to achieve on-demand and site-specific release of copper and iron ions. Chen et al. (72) engineered MetaCell—neutrophils loaded with NIR-thermo-responsive Fe–Cu MOF liposomes. Exploiting inflammatory chemotaxis, MetaCell accumulates in tumors, where 808 nm irradiation raises local temperature, ruptures liposomes and releases Fe–Cu MOF. The MOF-mediated synergy of ferroptosis and cuproptosis eradicated tumors without overt toxicity and conferred long-term survival, demonstrating the safety and immune-activating potential of an exogenously gated strategy.

Endogenous or exogenous responsive strategies offer an efficient, safe, and immune-synergistic paradigm for combined ferroptosis-cuproptosis in OSCC, while inspiring the design of next-generation multi-responsive carriers. However, tumor microenvironment heterogeneity may lead to uneven triggering and compromise efficacy stability. Therefore, the sensitivity and specificity of current responsive chemistries still require improvement, and the operability and safety of external stimuli in routine clinical settings remain to be fully validated.

### 5.3 Controlled drug release

Controlled drug release can prevent premature drug degradation and systemic exposure, enabling precise drug delivery at the target site while maintaining stable local concentrations and minimizing resistance escape. Zhang et al. (73) developed an injectable *in-situ* hydrogel (NCs-AS-ALG) composed of alginate cross-linked with Cu–Fe_3_O_4_ nanoclusters (NCs) and artesunate (AS). Gradual dissolution of NCs within tumor tissue releases Fe^2+^/Fe^3+^ and Cu^2+^, which react with AS to generate carbon-centered radicals (•C), amplify oxidative stress, deplete GSH, and down-regulate GPX4 and FDX1, thereby triggering ferroptosis and cuproptosis. Its translational potential to OSCC remains to be validated. Achieving sustained drug release and long-term concentration maintenance while simultaneously triggering ferroptosis-cuproptosis represents a pivotal strategy for future personalized and precise cancer therapy.

Controlled drug release holds great promise for post-operative adjuvant therapy or local recurrence control, particularly for precise intervention of residual lesions after surgery. It minimizes systemic toxicity to the greatest extent and provides an ideal strategy for achieving localized, long-acting treatment. Future research should focus on achieving personalized regulation of implant degradation rates and drug release kinetics, while large-scale preclinical safety studies should be conducted to provide data support for early-phase clinical trials.

### 5.4 Potential implications of combined therapies on patient outcomes

Although direct clinical evidence supporting combined ferroptosis and cuproptosis targeted therapy remains limited, combined ferroptosis- and cuproptosis-targeted strategies may hold potential to improve patient prognosis across multiple biological and clinical dimensions. In locally advanced disease, such combinatorial approaches may theoretically enhance treatment responses in preclinical models, which could provide a conceptual basis for less invasive interventions. In recurrent or metastatic disease, remodeling of the tumor immune microenvironment may potentially expand the population of patients who might benefit from immune checkpoint blockade. In platinum-resistant tumors, simultaneous inhibition of parallel cell death-escape pathways may help restore therapeutic responsiveness, thereby addressing the limitations of single-targeted approaches. Collectively, these potential clinical implications support further investigation and prioritization of this strategy in future translational and clinical trials.

## 6 Summary and prospects

### 6.1 Limitations and challenges

Despite the promising role of ferroptosis and cuproptosis in OSCC, the field faces significant hurdles before clinical translation can be realized. These challenges span mechanistic understanding, therapeutic design, and clinical validation.

Mechanistic Understanding Remains Fragmented. Current research often focuses on isolated “signature” molecules (e.g., GPX4, SLC7A11, FDX1) within genetically homogeneous cell lines, which fail to recapitulate the metabolic heterogeneity and complex stromal interactions of the *in vivo* tumor microenvironment (TME). A critical gap exists in systematically mapping the complete regulatory networks of these cell death pathways across different OSCC molecular subtypes, HPV statuses, or clinical stages. While crosstalk between ferroptosis and cuproptosis has been suggested, direct evidence identifying the key molecular integrators (e.g., how GSH depletion precisely tilts the balance between the two) and the ultimate cell fate decisions within a relevant TME is scarce. The over-reliance on simplified *in vitro* models raises a pivotal question: do these models capture the adaptive metabolic reprogramming that OSCC tumors employ *in situ* to evade metal ion toxicity? Advanced models, such as patient-derived organoids or heterotypic spheroids incorporating cancer-associated fibroblasts and immune cells, are urgently needed to bridge this translational gap.

Therapeutic Agents and Delivery Systems Require Optimization. Available inducers (e.g., erastin, RSL3, elesclomol) often suffer from poor pharmacokinetics, limited tumor selectivity, and narrow therapeutic windows, leading to potential off-target toxicity. NDDS offer a solution but introduce their own challenges: long-term biocompatibility, scalable manufacturing, cost-effectiveness, and comprehensive toxicology profiles are often inadequately addressed in proof-of-concept studies. Moreover, the field’s reliance on short-term cytotoxicity assays, which cannot substitute for formal safety evaluations, means most strategies remain at the pre-clinical stage. A key, yet underappreciated, challenge is adaptive resistance. Tumor cells can rapidly develop defenses through metabolic rewiring (e.g., enhanced glycolysis, upregulation of alternative antioxidant pathways), increased expression of metal-efflux pumps (e.g., ATP7A/B), or activation of compensatory survival signals (e.g., Nrf2, FSP1). Therapeutic strategies that do not pre-emptively account for these escape routes risk having their efficacy swiftly blunted. Furthermore, the complex and immunosuppressive tumor immune microenvironment also contributes to therapeutic resistance. Immune checkpoint molecules, tumor-associated macrophages, regulatory T cells, and myeloid-derived suppressor cells can attenuate the efficacy of ferroptosis and cuproptosis inducers, thereby forming a drug-tolerant microenvironment that promotes therapeutic resistance and early recurrence.

The Translational Gap is Pronounced. There is a striking lack of validation in large, well-annotated OSCC patient cohorts. The prognostic and predictive value of proposed biomarkers (e.g., expression of cuproptosis-related lncRNAs, immune phenotypes) remains largely correlative and uncertain. Critically, no standardized, quantitative, or dynamic assays exist for monitoring ferroptotic or cuproptotic activity in clinical settings. Are plasma or saliva levels of lipid peroxidation products (e.g., 4-HNE) or copper-chaperone proteins reliable, real-time indicators of therapy response? Prospective longitudinal studies are essential to answer this. Furthermore, the differential and potentially opposing effects of these death modalities on the immune microenvironment (ferroptosis as immunogenic vs. cuproptosis potentially immunosuppressive) complicate combination therapy design and necessitate sophisticated, context-dependent therapeutic sequencing.

### 6.2 Future directions

In conclusion, ferroptosis and cuproptosis represent two powerful, metabolically orchestrated forms of cell death that are intimately connected and dysregulated in OSCC. Their co-targeting, particularly through intelligent NDDS, is not merely additive but potentially synergistic, offering a promising avenue to dismantle multiple pillars of tumor survival simultaneously—metabolic adaptability, antioxidant defense, and immune evasion. The future of OSCC therapy may well lie in the precise spatiotemporal delivery of these “metallic bullets” to trigger an integrated cell death cascade.

Future efforts should leverage integrated multi-omics and single-cell sequencing to systematically map the ferroptosis-cuproptosis interactome in OSCC, identify master regulatory nodes, and stratify patients most likely to benefit from such therapies. Concurrently, next-generation intelligent nanocarriers designed to synergistically induce ferroptosis and cuproptosis in response to tumor-specific stimuli should be developed to achieve spatiotemporal synergy. Furthermore, the potential of traditional-medicine-derived active compounds, repurposed drugs, and de-novo synthetic small molecules to induce dual death should be thoroughly explored. In addition, future investigations should emphasize the rational combination of ferroptosis and cuproptosis inducers with immune checkpoint inhibitors or immune modulators to remodel the tumor immune microenvironment. Reversing immunosuppression and restoring effective antitumor immune surveillance will help eliminate residual and drug-resistant tumor clones, thereby enhancing treatment durability and achieving more comprehensive therapeutic benefits for OSCC.

Given the superficial and accessible location of oral lesions, localized delivery of ferroptosis and cuproptosis modulators offers a promising strategy to avoid systemic toxicity. Future research should focus on developing injectable intralesional systems, mucoadhesive topical formulations, and intraoperative implantable devices for precise, site-specific induction of regulated cell death. These local approaches can achieve high intratumoral drug concentrations while reducing off-target effects, in line with the principles of precision medicine.

**Figure 4:**
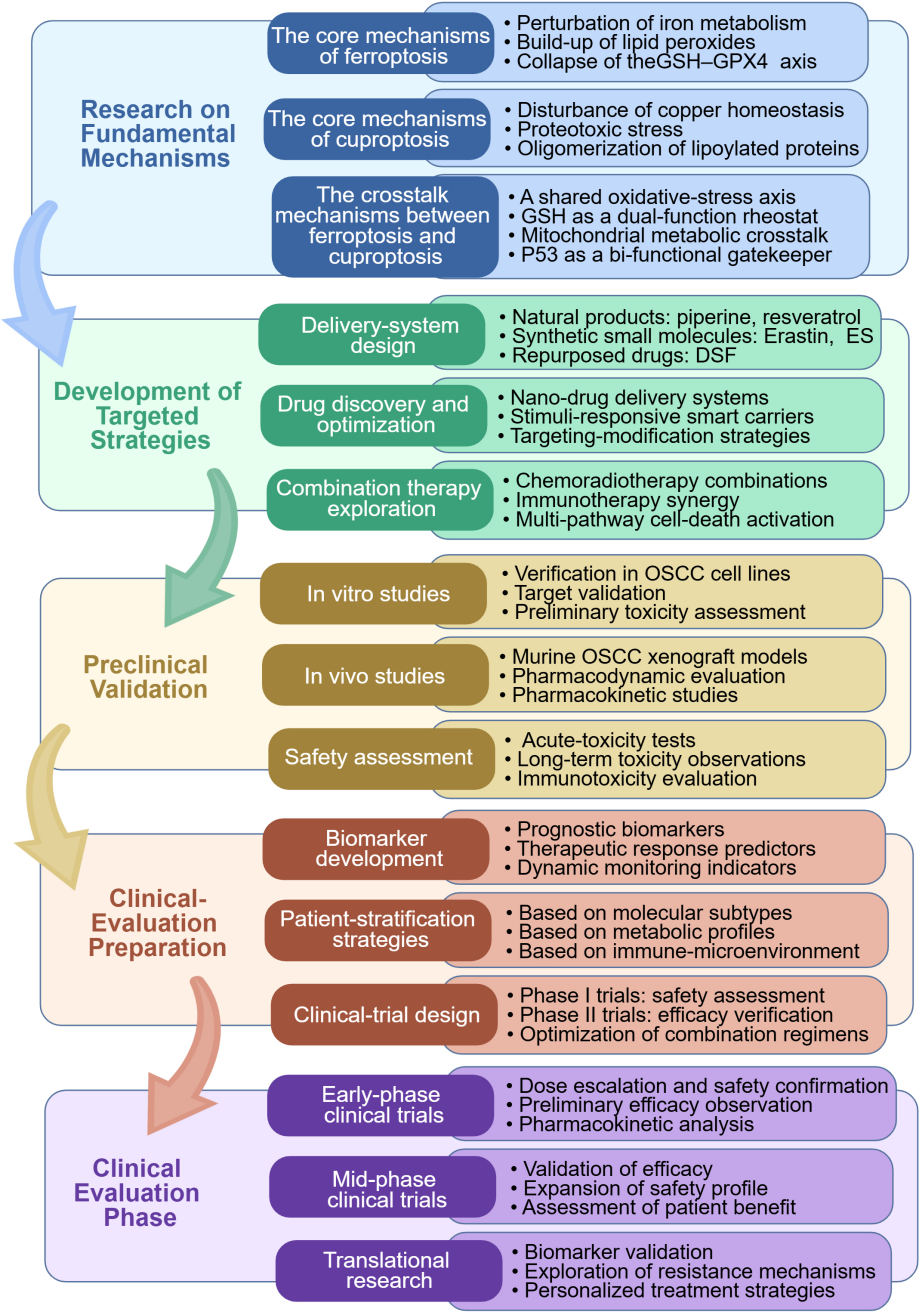
Flowchart of therapeutic strategies and clinical translation targeting ferroptosis/cuproptosis in oral squamous cell carcinoma. Created with BioGDP.com, cited in (74).

In clinical translation, robust multi-center preclinical studies with large sample sizes are essential to verify drug efficacy and safety. Subsequently, early-phase clinical trials incorporating validated biomarkers and adaptive designs should be initiated to evaluate their therapeutic value in OSCC patients. A proposed translational roadmap integrating these strategies is summarized in **Figure 4**. By deepening mechanistic insights, integrating advanced technologies, and rigorously advancing clinical evaluation, ferroptosis-cuproptosis-based therapies hold great promise for evolving into a precise, potent, and low-toxicity treatment strategy for OSCC.
